# Air Pollution Monitoring Design for Epidemiological Application in a Densely Populated City

**DOI:** 10.3390/ijerph14070686

**Published:** 2017-06-25

**Authors:** Kyung-Duk Min, Ho-Jang Kwon, KyooSang Kim, Sun-Young Kim

**Affiliations:** 1 Department of Public Health Science, Graduate School of Public Health, Seoul National University, Seoul 08826, Korea; fortop@snu.ac.kr; 2 Department of Preventive Medicine, Dankook University College of Medicine, Cheonan 31116, Korea; hojang@dankook.ac.kr; 3 Department of Occupational Environmental Medicine, Seoul Medical Center, Seoul 02053, Korea; kyoosang@daum.net; 4 Institute of Health and Environment, Seoul National University, Seoul 08826, Korea

**Keywords:** air pollution, fine particulate matter, monitoring design, site selection spatial variability

## Abstract

*Introduction*: Many studies have reported the association between air pollution and human health based on regulatory air pollution monitoring data. However, because regulatory monitoring networks were not designed for epidemiological studies, the collected data may not provide sufficient spatial contrasts for assessing such associations. Our goal was to develop a monitoring design supplementary to the regulatory monitoring network in Seoul, Korea. This design focused on the selection of 20 new monitoring sites to represent the variability in PM_2.5_ across people’s residences for cohort studies. *Methods*: We obtained hourly measurements of PM_2.5_ at 37 regulatory monitoring sites in 2010 in Seoul, and computed the annual average at each site. We also computed 313 geographic variables representing various pollution sources at the regulatory monitoring sites, 31,097 children’s homes from the Atopy Free School survey, and 412 community service centers in Seoul. These three types of locations represented current, subject, and candidate locations. Using the regulatory monitoring data, we performed forward variable selection and chose five variables most related to PM_2.5_. Then, k-means clustering was applied to categorize all locations into several groups representing a diversity in the spatial variability of the five selected variables. Finally, we computed the proportion of current to subject location in each cluster, and randomly selected new monitoring sites from candidate sites in the cluster with the minimum proportion until 20 sites were selected. *Results*: The five selected geographic variables were related to traffic or urbanicity with a cross-validated *R*^2^ value of 0.69. Clustering analysis categorized all locations into nine clusters. Finally, one to eight new monitoring sites were selected from five clusters. *Discussion*: The proposed monitoring design will help future studies determine the locations of new monitoring sites representing spatial variability across residences for epidemiological analyses.

## 1. Introduction

Many cohort studies have found associations between long-term exposure to air pollution and various health endpoints by employing air pollution data from regulatory monitoring networks operated by governments [1,2]. However, these regulatory monitoring networks were designed primarily to monitor air quality and regulate pollution sources, rather than to evaluate the health effects of air pollution. Thus, air pollution measurements collected in regulatory monitoring networks may not sufficiently represent the variability of air pollution concentrations across people’s residences.

To gauge air pollution variability representative of residences, some studies have carried out project-based monitoring campaigns independent of or supplementary to regulatory monitoring networks. These campaigns, mostly performed in urban areas of the USA and Europe, established monitoring sites at public offices, schools, participant homes, and/or busy roads [3,4,5,6]. However, these studies did not provide detailed methodologies for their monitoring designs, including the determination of the numbers and/or locations of new monitoring sites.

A few studies elaborately developed methodologies for monitoring designs focusing on the selection of monitoring sites given a fixed number of sites. Studies in Toronto, Canada and Iowa City, Iowa, USA, introduced site selection approaches based on location–allocation models. These designs selected new sites that provided the maximized spatial variability of predicted concentrations for PM_10_ and NO_2_ in surrounding areas, in addition to high population density [7,8]. However, their designs did not incorporate existing regulatory monitoring sites, which are a good resource to combine with the new sites.

In this study, our goal was to develop a monitoring design supplementary to a regulatory monitoring network for representing the spatial variability of PM_2.5_ across people’s residences for epidemiological studies in Seoul. As a highly populated capital city of the Republic of Korea with approximately 10 million people in an area of 605.25 km^2^, Seoul serves as a good example for developing an effective monitoring design that will help predict individual-level air pollution concentrations, and to assess the resulting health effects. Our design specifically focused on the selection of 20 new monitoring sites—the minimum number of sites possible, given logistical and financial constraints. We selected PM_2.5_, as an example, based on previous studies showing an association with human health, and fine-scale spatial variability largely affected by anthropogenic pollution sources [2,9].

## 2. Materials and Methods

### 2.1. Data Collection and Processing

#### 2.1.1. Air Pollution Monitoring Data

We obtained hourly PM_2.5_ measurements at 37 sites in Seoul from the National Institute of Environmental Research [10], and computed the annual average concentrations of PM_2.5_ at each site. The Ministry of Environment (MOE) in the Republic of Korea operated 294 regulatory air pollution monitoring sites in 2010 on a national scale. In Seoul, the 37 sites included 25 urban background and 12 urban roadside sites. The 25 urban background sites were located on roof tops of municipal buildings without any dominant nearby pollution sources for monitoring air pollution exposure levels in the population. Each of the 25 districts in Seoul had one urban background site in 2010. The 12 urban roadside sites were located next to busy roads, to assess air pollution emitted from traffic. Using the hourly measurements, we computed daily average concentrations for days with more than 18 hourly measurements (75%), and then computed representative annual averages at all sites. All 37 sites met our site inclusion criteria; at least one daily average per month for more than 9 months, no more than 91 missing days (25%), and less than 45 consecutive missing days [11].

#### 2.1.2. Location Data

We used three types of location data for our monitoring design. The three types included regulatory monitoring sites (“current location”), residences (“subject location”), and community service centers (“candidate location”). The addresses and coordinates of the 37 regulatory monitoring sites in Seoul were obtained from the Annual Report of Ambient Air Quality in Korea 2010 [10]. For residences, we obtained 31,097 children’s home addresses from the Atopy Free School survey in 2010 [12]. These children, under the age of 13, joined the survey based on their elementary schools and daycare centers distributed over the 25 districts in Seoul, which largely represent the locations of Seoul residents. Lastly, we obtained the addresses of 412 community service centers, out of a total of 422 in Seoul, from center websites, as candidates for new monitoring sites. Ten centers where current regulatory monitoring sites were already located, were excluded. Addresses of children’s homes in the Atopy Free School survey and community service centers were geocoded using geocoding software, GeoCoder-Xr (3.0, Geoservice, Seoul, Korea). 

#### 2.1.3. Geographic Variables

We computed 313 geographic variables at current, subject, and candidate locations. These variables represented potential air pollution sources for eight categories including traffic, demographic characteristics, land use, transportation facilities, physical geography, emissions, vegetation and altitude (Table S1). All source data were collected or generated in 2010, except land use data, which were generated in 2007, and updated for some areas in 2009. The details on their relationships with air pollution and computation procedure were published elsewhere [13]. The variables were computed as two types of metrics: proximity and density. Proximity variables were computed as the distances closest to pollution sources, such as major roads, airports/ports, and coastline. Density variables were the sums of entities or percentage of areas within circular buffers, applied to road networks, population, and land use.

We recoded and excluded some geographic variables to better reflect relationships with air pollution and/or to obtain sufficient spatial variability. All proximity variables were truncated at 1 km—or 2 km for coastline, river, and northern borderline—and log transformed. In addition, we excluded 40 variables with less than 10% unique values and less than 10% buffer areas attributed to each land use.

### 2.2. Air Pollution Monitoring Design

**Figure 1:**
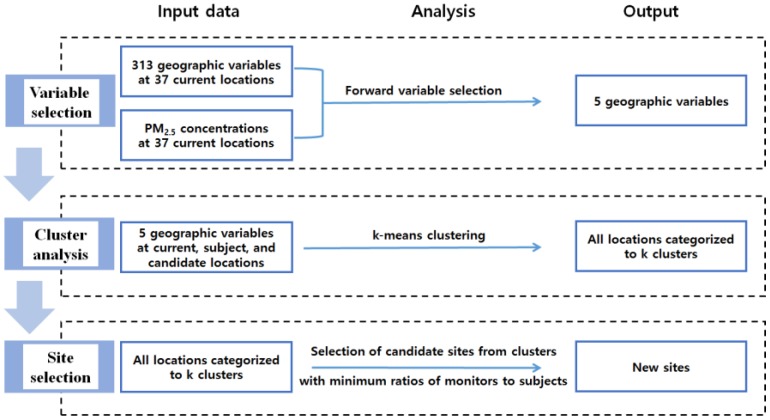
The procedure of selecting 20 new monitoring sites supplementary to regulatory monitoring sites in Seoul, Korea.

We selected 20 candidate locations, where PM_2.5_ concentrations were poorly represented by current locations, in terms of distribution of related geographic variables. This approach was based on our assumption that PM_2.5_ annual average concentrations are largely determined by a limited set of geographic variables. The associations of geographic variables with PM_2.5_ have been well reported in previous studies across different cities and countries [14,15,16,17]. Many of these studies employed land use regression, which regresses air pollution concentrations on a subset of geographic variables, selected out of a large number of variables by model selection procedures combining statistical techniques and scientific choices [18]. Our monitoring design for selecting new sites consisted of three steps: variable selection, cluster analysis, and site selection (Figure 1). Forward variable selection and k-means clustering used for the design were implemented in R version 3.2.3.

#### 2.2.1. Variable Selection

We chose five geographic variables most related to PM_2.5_ annual average concentrations across 37 regulatory monitoring sites using a forward selection approach. The forward selection procedure starts with a null model, and proceeds by adding variables one at a time, to maximize the explained variability, until none of the remaining variables are significant. We restricted the maximum number of variables to five, given the limited number of monitoring sites. To prevent multicollinearity, variance inflation factors (VIF) and Pearson correlation coefficients between two variables were investigated. We removed an added variable if the VIF value exceeded 10, or the correlation coefficient was greater than 0.7 with any of the selected variables. We evaluated the models using leave-one-out cross-validation (LOOCV). In LOOCV, we left one site out, fitted the model using the data at remaining sites, and made a prediction at the left-out site. After repeating this procedure for the remaining 36 sites, we obtained cross-validated predictions at all 37 sites. Then, we computed the cross-validated *R*-squared value (*R*^2^), which is one minus the mean square error (MSE) divided by data variance. This MSE-based *R*^2^ compares predictions to observations based on the identity line [19,20].

#### 2.2.2. Cluster Analysis

Using k-means clustering, we categorized all three types of the 38,680 locations into k groups representing contrasts in the spatial variability of the five selected geographic variables. We scaled all variables by subtracting means and dividing by standard deviations, to avoid the large impact of unit differences across variables on the analysis. K-means clustering is an iterative algorithm that defines k cluster centers randomly given the number of clusters (k), assigns observations with multiple dimensions to k clusters based on the shortest distance to the centers, and re-computes cluster centers of groups, leading to regrouping of observations until all observations are classified into the same clusters as in the previous regrouping [21].

K-means clustering has been widely used given its easy implementation and computational effectiveness [22,23]. However, selection of initial cluster centers and pre-specification of the number of clusters were indicated as major challenges [24,25]. To find the best solution for the initial definition of cluster centers, we repeated our analysis 1000 times with 1000 different initial cluster centers. For each analysis, we computed the sum of within-cluster sum of squared errors (SSW), which is the sum of squared differences from the cluster means. The analysis that provided the lowest sum of SSW, indicating minimized within-cluster heterogeneity, was considered the best solution.

We determined the number of clusters using the decrease in overall deviation (DiD) [25]. DiD is the percent change in the average of SSW relative to total sum of squared errors (SSE) over characteristics (Equation (1)). As the number of clusters increases, within-cluster variability relative to total variability decreases, resulting in an increase in DiD, and then a plateau that reflects the minimization of overall deviation. We computed DiDs for 1–50 clusters, and chose the optimal number of clusters based on k at the beginning of the plateau. For characteristics of DiD, we used predicted PM_2.5_ annual average concentrations, in addition to geographic variables, at all three location types, and determined the optimal number which was consistent between the two characteristics. PM_2.5_ annual average predictions were calculated by using regression coefficients of land use regression models and five selected geographic variables at all locations.

(1) $$DiD\left(\%\right)=100\times \left(1-\frac{1}{J}\overset{J}{\underset{j=1}{\sum }}\overset{K}{\underset{k=1}{\sum }}\frac{SSW_{jk}}{SSE_{j}}\right)=100\times \left(1-\frac{1}{J}\overset{J}{\underset{j=1}{\sum }}\overset{K}{\underset{k=1}{\sum }}\overset{n_{k}}{\underset{i=1}{\sum }}\frac{{\left(Y_{ijk}-{\bar{Y}}_{jk}\right)}^{2}}{{\left(Y_{ijk}-{\bar{Y}}_{j}\right)}^{2}}\right)$$

*i* = observation in the cluster k (1 to *n_k_*); *j* = characteristic (1 to *J*); *k* = cluster (1 to *K*); *J* = 1 and 5 for PM_2.5_ predictions and five geographic variables, respectively.

To understand the characteristics of each cluster, we produced heatmaps that illustrate the mean of each of the five scaled geographic variables across the three types of locations included in each cluster. Heatmaps allowed the comparison of the distributions of geographic variables across clusters.

#### 2.2.3. Site Selection

Given all locations categorized into k groups, we calculated the proportion of the number of current locations to that of subject locations in each cluster. We then randomly selected a new monitoring site from candidate sites in the cluster with the minimum proportion of current to subject locations. The computation of the proportion and the selection of a new site were repeated until 20 sites were completed.

#### 2.2.4. Sensitivity Analysis

To evaluate the robustness of our clustering results, we repeated k-means clustering 100 times using 90% of the locations, after randomly excluding 10%, and compared the results to that of the original analysis using all locations. For this comparison, we computed the Rand index, which summarizes the agreement or disagreement of a pair of observations between two categorization methods, as a measure of agreement. Each pair could be either a matched pair (assigned to the same cluster) or an unmatched pair (assigned to different clusters) in a cluster analysis. This classification can be either the same or different from another cluster analysis. Agreement was defined as two matched or two unmatched pairs in both analyses using 90% of all locations, whereas disagreement was characterized by a matched and an unmatched pair in either analysis. The Rand index was the proportion of the number of pairs in agreement to the number of all pairs. In this study, the adjusted Rand index, corrected for random chance of agreement, was employed. Adjusted Rand indices higher than 0.90, 0.80 and 0.65 indicate excellent, good, and moderate agreement, respectively [25].

In addition, we applied our design to another pollutant, nitrogen dioxide (NO_2_), to determine whether the design performed well for pollutants with different characteristics. NO_2_ has been of particular interest as a traffic-related pollutant, with fine-scale spatial variability, resulting in adverse health effects [1,18].

## 3. Results

### 3.1. Distributions of Locations and Air Pollution Concentrations

#### 3.1.1. Three Types of Locations

**Figure 2:**
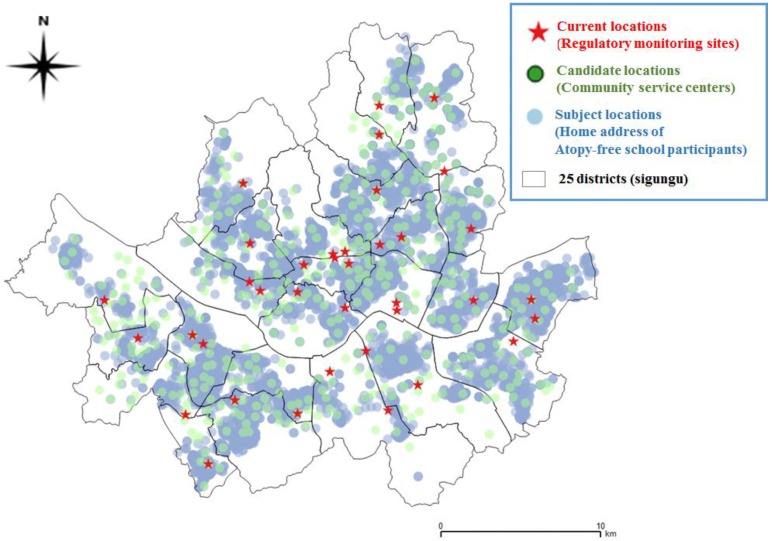
Map of 37 current locations (regulatory monitoring sites), 412 candidate locations (community service centers), and 31,097 subject locations (home addresses of the Atopy Free School survey children) in Seoul, Korea.

Figure 2 shows the locations of 37 regulatory monitoring sites, 31,097 Atopy Free School survey children’s homes, and 412 community service centers corresponding to “current”, “subject”, and “candidate” locations, respectively, in Seoul. The current locations were evenly distributed over the city, because each of the 25 districts includes at least one regulatory monitoring site. Some subject locations were far from their current locations.

#### 3.1.2. Annual Average Concentrations of PM_2.5_

Table S2 shows summary statistics of PM_2.5_ annual average concentrations in 2010 at 37 regulatory monitoring sites in Seoul. The means of the annual average concentrations were 26.8 (standard deviation (SD) = 3.7) μg/m^3^ across 37 regulatory monitoring sites. PM_2.5_ concentrations at the 25 urban monitoring sites (mean = 24.9, SD = 1.8 μg/m^3^) were lower and less variable than at the 12 urban roadside sites (30.6, 3.8 μg/m^3^).

### 3.2. Air Pollution Monitoring Design

#### 3.2.1. Variable Selection

Table 1 lists the five selected variables used for PM_2.5_ annual average concentrations in 2010 in Seoul. The five selected variables for PM_2.5_ were the sum of road lengths for major roads within 100 m, the proportion of water surface land use within 500 m, the number of construction companies within 1 km, the distance to the nearest bus stop, and the number of construction workers within 100 m. The proportion of water surface land use possibly represents traffic, because two out of the nine highways in Seoul were constructed alongside the Han River, which runs through the middle of Seoul. Two traffic-related variables showed the strongest relationships with PM_2.5_. The sum of road lengths for major roads, the proportion of water surface land use, and the number of construction companies were positively associated with PM_2.5_, whereas the distance to the nearest bus stop, and the number of workers in construction were negatively associated. The LOOCV *R*^2^ was 0.69 (Figure S1).

Means and standard deviations for the selected geographic variables across three types of locations are shown in Table S3. There was a noticeable difference in the sum of lengths for major roads between current locations, and subject and candidate locations, possibly because current locations included urban roadside sites located next to busy and large roads.

#### 3.2.2. Cluster Analysis

**Figure 3:**
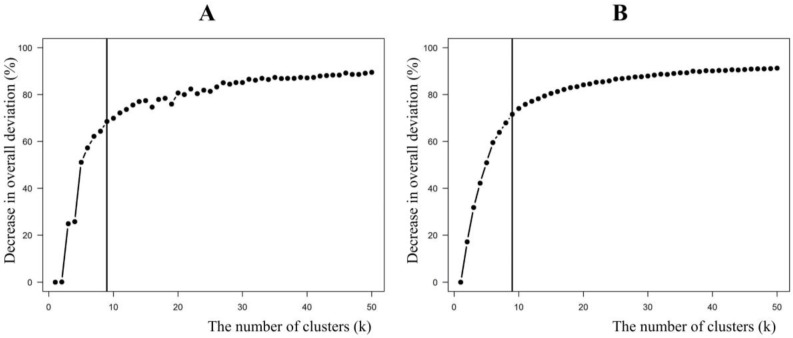
Decrease in overall deviation (DiD) based on predicted PM_2.5_ concentration (**A**) and five geographic variables (**B**) against the numbers of clusters (k) (vertical lines indicate nine clusters).

Figure 3 displays the increasing trend in DiD as the number of clusters increases. We chose nine as the optimal number of clusters, where the rates of increase in DiDs based on the five geographic variables, as well as PM_2.5_ predictions, became prominently consistent.

Distributions of the numbers of current and subject locations varied across clusters (Table 2). Cluster 3 consisted of the largest numbers of subject locations (47.9%) and current locations (43.2%). However, 16 out of 37 monitoring sites may not sufficiently represent the subject locations. Clusters 4 and 5 had the smallest portions of subject locations (0.1% and 0.6%, respectively), also with current location values of zero or one. On the contrary, clusters 7–9 included none or very few current locations compared to many subject locations, suggesting the need for new monitoring sites.

The heatmap in Figure S2 shows different patterns of five geographic variables across clusters. The mean of the scaled sum of road lengths was uniquely large at locations in cluster 6 and the mean proportion of water surface land use in the cluster 5 was larger than other clusters. All current locations in these clusters were urban roadside sites (one for cluster 5 and three for cluster 6), indicating that the locations in the clusters were largely affected by traffic. The locations in cluster 3, with the largest portion of subject locations, showed larger mean distances to bus stops and smaller means of traffic and urban land use variables, possibly indicating residential areas. Clusters 7–9, with many subject locations but relatively few monitoring sites, all showed relatively little impact from traffic-related variables.

#### 3.2.3. Site Selection

Clusters 2 and 7 did not include any current locations, leading to the lowest proportions of current to subject locations. Clusters 3, 8 and 9 also showed relatively low proportions of 3–16 current locations to 3567–14,888 subject locations. We selected one new site from the candidate sites in each of the clusters 2 and 9, and four, six, and eight sites in clusters 7, 3 and 8, respectively (Table 2). Figure S3 shows the spatial distribution of the 20 new sites, out of the 412 candidate sites in Figure S4. The addition of new sites increased variability of some geographic variables compared to the variability across current locations only (Figure S5). In addition, predicted PM_2.5_ at new sites covered a low range of predicted PM_2.5_ at subject locations, which was not represented by current locations. This pattern indicates good representation of PM_2.5_ variability across residences, when new sites were added to current monitoring sites (Figure S6).

#### 3.2.4. Sensitivity Analysis

K-means clustering using 100 sets of 90% locations gave the average adjusted Rand index of 0.93 (range = 0.51–0.99). Ninety-three percent of the indexes were greater than 0.9, whereas 7% were less than 0.65, indicating excellent agreement. Our monitoring design including variable selection, clustering analysis, and site selection was well applied to NO_2_ (Table 3, Tables S4 and S5, and Figures S7–S10).

## 4. Discussion

We developed an air pollution monitoring design for PM_2.5_ in Seoul, Korea, for the purpose of representing the spatial variability of exposure across people’s residences for application to epidemiological studies. This design specifically focused on the selection of new monitoring sites to supplement existing regulatory monitoring sites. We established a design consisting of three procedures to achieve our goal: the selection of geographic variables most related to PM_2.5_, the spatial clustering of selected geographic variables largely represented by residential locations, and the determination of candidate sites as new monitoring sites with geographic features dominant across residences but underrepresented by existing monitoring sites.

We leveraged more than 30,000 residential locations to represent the spatial variability of geographic features related to PM_2.5_ concentrations for Seoul residents. Previous studies of air pollution monitoring designs tended to rely heavily on monitoring data. For example, two previous studies in Canada and the United States developed monitoring designs for selecting new monitoring sites in city areas based on geographic variables to characterize the spatial variability of NO_2_ and PM_10_ [7,8]. Using land use regression of selected geographic variables on air pollution concentrations from regulatory monitoring networks or project-based mobile sampling, they created exposure surfaces of predicted air pollution concentrations over city areas. Assuming that the prediction surfaces are the true concentration surfaces, they selected locations of new monitoring sites where there was high variability of predicted concentrations within a surrounding area and large population. However, this assumption would not hold when regulatory monitoring networks do not sufficiently represent residential locations. In particular, limited numbers of regulatory monitoring sites (16 and 67 in the two studies) may not be sufficient to characterize residential locations.

The development of a monitoring design that correctly represents people’s exposures is important for subsequent health analyses. A previous simulation study showed that exposure prediction relying on monitoring network data produced an exposure measurement error in predicted exposures at people’s residences when monitored locations do not represent population locations [26]. This measurement error resulted in biased and/or imprecise health effect estimates in subsequent health analyses [27,28,29]. Given our ultimate goal of utilizing our monitoring design for health analyses, the design used a large amount of geographic information across residential locations, instead of the relationships captured by monitoring data.

The five geographic variables selected in our design largely reflected metropolitan characteristics of Seoul, and were directly or indirectly related to pollution sources, particularly for traffic. The positive coefficient with the largest magnitude for increment from the 10th to the 90th percentiles of the sum of major road length variable would reflect traffic as one of the major pollution sources of PM_2.5_ in Seoul. A proportion of water surface land use, indicating proximity to metropolitan highways, was also positively and strongly related to PM_2.5_ concentrations. This relationship reflected the impact of metropolitan highways constructed alongside the Han River which flows through Seoul (Figure S11). The strong associations of traffic variables could be due in part to the large contribution of urban roadside sites, totaling about one-third of all sites, to our land use regression. Clusters 5 and 6, that showed the dominant influence of two traffic-related variables (Figure S2), included few subject locations. However, clusters 1 and 7, with relatively larger impact of traffic variables consisted of some subject locations, suggesting residences located close to traffic. In Seoul, median distance from subject locations to the nearest major roads was 256.9 m. The proximity of many residential locations in Seoul to major roads possibly results from people’s preference for residences that are easily accessible to transportation in the densely populated metropolitan area with heavy traffic. Five geographic variables that showed good predictive ability for air pollution concentrations at monitoring sites may be too limited to predict those at people’s residences. However, a previous study comparing land use regression and partial least squares (PLS) regression, a dimension reduction approach, showed largely consistent predictions for PM_10_ and NO_2_ at centroids of residential census tracts in South Korea. Their land use regression included six variables, whereas PLS provided summary predictors estimated from 300 variables [30].

The regression coefficients of geographic variables generally showed anticipated directions. The sum of major road lengths and the proportion of water surface, representing traffic density, showed positive associations with PM_2.5_, whereas the distance to the bus stop gave a negative association. The number of construction companies within 1 km was also positively associated. This variable would mean commercial and developed areas, given its inclusion of site offices as well as head offices of the construction industry, possibly located in the central part of the city. The only variable showing a relationship different from the anticipated direction was the number of construction workers within 100 m. This would reflect other information than the original, because the construction-related land use variable was already included and/or the small buffer size of 100 m could not sufficiently reflect such land use. Instead, this variable may represent fine local environments such as proximity to roads, bus stops, or subway stations which would be negatively associated with PM_2.5_.

Our design provides practicable suggestions to select candidate sites that supplement existing monitoring networks. Our design, however, could be applied to areas without any existing monitors, when we import the relationship between geographic variables and air pollution from other areas with similar environments. This design could also be utilized to locate temporally fixed and/or rotating sites in project-based monitoring campaigns focusing on specific cohort participants. In addition, we used community service centers as candidates for new sites, because they are largely located in densely-populated residential areas and are easy to collaborate with regarding public health concerns, such as air pollution in communities. Other public buildings or census-based centroids could be alternative options.

One of the key limitations of our study is our use of k-means clustering, which could lead to results that are sensitive to the choice of the number of clusters and initial cluster centers. To find the best solution, we selected the number of clusters and initial cluster centers that minimized within-cluster variability used in a previous study of air pollution [25]. Other methods, such as the silhouette method [31] or information-theoretic approach [32], could also be employed. However, our sensitivity analysis showed largely consistent categorization of the locations to the original. It should also be noted that our intention of using k-means clustering was to guide us to partition locations with sufficient within-cluster similarity and between-cluster difference in geographic features, rather than to identify the most accurate classification. We selected new sites randomly within each cluster without considering other information. Alternatively, future studies could consider prioritizing a site located in largely populated areas, or distant from another selected site, and/or regulatory monitoring sites. As another limitation, we assumed that children’s homes from the Atopy Free School survey represented residential locations in Seoul. This survey recruited children based on schools from the 25 districts of Seoul, and provided rich spatial data with a large number of residential locations, particularly for children as a population vulnerable to air pollution [33]. However, it is possible that there are groups of residents whose locations were not represented by this survey. Future research needs to use different location data to assess the representativeness of our design for the general population in Seoul. Our design did not incorporate wind direction, which would affect very different air pollution concentrations between sites upwind and downwind of a road. However, since previous studies reported inconsistent wind direction over a year in Seoul [34], it is less likely that the long-term air pollution concentrations, on which our design focused, were affected. Finally, we focused on a spatial monitoring design to represent the spatial variability of air pollution. There have been recent interests in mobile or personal monitoring that characterizes spatially and temporally varying air pollution using vehicles and/or low-cost sensors [35]. Future studies should develop monitoring designs that guide the selection of new sites in space and time to represent spatiotemporal patterns of air pollution.

## 5. Conclusions

We developed a monitoring design that is applicable to a new regulatory monitoring design to characterize residential air pollution exposure in urban areas. This design will allow us to improve exposure prediction models and to assess the health effects of air pollution.

## Figures and Tables

**Table 1:** Five selected geographic variables and cross-validated *R*^2^s from land use regression of PM_2.5_ annual average concentrations during 2010 in Seoul, Korea.

Variable	β ^a^	*p* Value	LOOCV *R*^2^
Length of major road ^b^ (100 m buffer)	3.58	<0.001	0.69
Proportion of water surface land use (500 m)	0.67	<0.001	
Number of construction companies (1000 m)	3.01	0.001	
Distance to the nearest bus stop	−2.46	0.013	
Number of employees in construction industries (100 m)	−1.91	0.025	

^a^ Estimated regression coefficient multiplied by an increment (90th–10th percentile) of each variable; ^b^ Major road defined as all national and metropolitan highways, and local roads with more than six lanes.

**Table 2:** Numbers (%) of subject, current, and candidate locations, proportions of current to subject locations, and numbers of new selected sites across nine clusters for PM_2.5_.

	Cluster 1	Cluster 2	Cluster 3	Cluster 4	Cluster 5	Cluster 6	Cluster 7	Cluster 8	Cluster 9	Total
Current ^a^	9 (24.3)	0 (0.0)	16 (43.2)	0 (0.0)	1 (2.7)	3 (8.1)	0 (0.0)	3 (8.1)	5 (13.5)	37 (100)
Subject ^b^	2587 (8.3)	505 (1.6)	14,888 (47.9)	34 (0.1)	187 (0.6)	303 (1.0)	2246 (7.2)	6780 (21.8)	3567 (11.5)	31,097 (100)
Candidate ^c^	60 (14.6)	19 (4.6)	131 (31.8)	0 (0.0)	4 (1.0)	7 (1.7)	25 (6.1)	136 (33.0)	30 (7.3)	412 (100)
Current/Subject ^d^	34.8	0	10.8	0	53.5	99.0	0	4.4	14.0	
New sites		1	6				4	8	1	

^a^ Current location: regulatory air pollution monitoring sites; ^b^ Subject location: home addresses of the Atopy Free School survey children; ^c^ Candidate location: community service centers; ^d^ Proportion of the number of current locations to that of subject locations, multiplied by 10^4^.

**Table 3:** Numbers (%) of subject, current, and candidate locations, proportions of current to subject locations, and numbers of new selected sites across nine clusters for NO_2_.

	Cluster 1	Cluster 2	Cluster 3	Cluster 4	Cluster 5	Cluster 6	Cluster 7	Cluster 8	Total
Current ^a^	2 (5.4)	0 (0.0)	0 (0.0)	0 (0.0)	21 (56.8)	1 (2.7)	1 (2.7)	12 (32.4)	37 (100)
Subject ^b^	228 (0.3)	2 (3.5)	2131 (0.6)	1577 (9.4)	20,935 (63.3)	3336 (6.8)	187 (4.5)	2701 (8.1)	31,097 (100)
Candidate ^c^	5 (1.2)	0 (0.0)	33 (8.0)	20 (4.9)	247 (60.0)	32 (7.8)	4 (1.0)	71 (17.2)	412 (100)
Current/Subject ^d^	87.72	0	0	0	10.03	3.00	53.48	44.43	
New sites			4	3	9	4			

^a^ Current location: regulatory air pollution monitoring sites; ^b^ Subject location: home addresses of the Atopy Free School survey children; ^c^ Candidate location: community service centers; ^d^ Proportion of the number of current locations to that of subject locations, multiplied by 10^4^.

## Supplementary Materials

The following are available online at www.mdpi.com/1660-4601/14/7/686/s1, Table S1: List of geographic variables in eight categories with their data sources and types of data, Table S2: Summary statistics of annual average concentrations for PM_2.5_ (μg/m^2^) in 2010 at 37 regulatory monitoring sites in Seoul, Korea, Table S3: Means and standard deviations of the five selected geographic variables from land use regression of PM_2.5_ annual average concentrations during 2010 across current, subject and candidate locations in Seoul, Korea, Table S4: Means and standard deviations of the five selected geographic variables from land use regression of NO_2_ annual average concentrations during 2010 across current, subject and candidate locations in Seoul, Korea, Table S5: Five selected geographic variables and cross-validated *R*^2^*s* from land use regression of NO_2_ annual average concentrations during 2010 in Seoul, Korea, Figure S1: Scatter plot of observed and cross-validation predicted annual average concentrations of PM_2.5_ across 37 regulatory monitoring sites during 2010 in Seoul, Korea (leave-one-out cross-validation *R*^2^ of 0.69), Figure S2: Heatmap of the five geographic variables at current, subject, and candidate locations across nine clusters for PM_2.5_, Figure S3: Map of 20 selected new monitoring sites for PM_2.5_ along with current, candidate, and subject locations in Seoul, Korea, Figure S4: Maps of candidate sites in each of the nine clusters determined from cluster analysis for PM_2.5_ (**A**) and new selected sites with regulatory monitoring sites (**B**), Figure S5: Distributions of five scaled geographic variables across subject locations (left), current locations (middle), and new sites (right) selected from candidate locations by using the monitoring design for PM_2.5_, Figure S6: Variability of predicted PM_2.5_ across subject locations (left), current locations (middle), and new sites (right), Figure S7: Scatter plot of observed and cross-validation predicted annual average concentrations of NO_2_ across 37 regulatory monitoring sites during 2010 in Seoul, Korea (leave-one-out cross-validation *R*^2^ 0.58), Figure S8: Decrease in overall deviation (DiD) based on predicted NO_2_ concentration (left) and five geographic variables (right) against the numbers of clusters (k) (vertical lines indicating nine clusters), Figure S9: Heatmap of the five geographic variables at current, subject, and candidate locations across eight clusters for NO_2_, Figure S10: Map of 20 selected new monitoring sites for NO_2_ along with current, candidate, and subject locations in Seoul, Korea, Figure S11: Map of Han River and metropolitan and national highways in Seoul.
