# Supplementary material for: Air Pollution Monitoring Design for Epidemiological Application in a Densely Populated City
Source: Int J Environ Res Public Health. 2017 Jun 25;14(7):686. doi: 10.3390/ijerph14070686 (PMC5551124; doi:10.3390/ijerph14070686)
Supplement: Supplementary file 1 [file ijerph-14-00686-s001.pdf]

International Journal of
Environmental Research and Public Health

MDPI

Air Pollution Monitoring Design for Epidemiological Application in a Densely Populated City

Kyung-Duk Min, Ho-Jang Kwon, KyooSang Kim and Sun-Young Kim

Supplementary Materials

Table S1. List of geographic variables in eight categories with their data sources and types of data.

Category [a]	Variable	Source	Type of data (data format)
Traffic	Distance to the nearest roads (all roads, highway, and MR)	KTDB	Road network (line)
	Sum of road lengths (all roads, highway, and MR)[b]		
	Number of registered vehicles	KOSIS	Vehicle registration (attributable table)
Demographic characteristics	Number of people	SGIS	Census (attributable table)
	Number of households		
	Numbers of housing buildings by a type of residence and by a constructed year		
	Numbers of companies and employees by a type of business		
Land use	Proportions of residential, industrial, commercial, cultural, transportation, public facility, agricultural, forest, grassland, wetland, bare ground, and water areas	EGIS	Land cover map (polygon)
Transportation facilities	Distances to the nearest railroad and subway station	SGIS	Railroad and subway stations (point)
	Distance to the nearest bus stop	Biz-GIS	Bus stop (point)
	Distance to the nearest air port	ODP	Airport (table)
	Distance to the nearest major port	SP-IDC	Port (table)
Physical geography	Distance to river	SGIS	River (polygon)
	Distance to coastline	NSIC	Coastline (line)
	Distance to the military demarcation line	SGIS	Administrative boundary (polygon)
Emissions	Proportions of major pollutants (CO, NOx, SOx, TSP, PM10, VOC, and NH3)	NIER	Emission estimates (attributable table)
Vegetation	Annual summary (average, minimum, and maximum) of NDVI	IIS	Satellite image (raster)
	Median value in August for previous, current and following years		
Altitude	Absolute elevation	USGS	Digital Elevation Data (raster)
	Proportion of concentric elevation points above or below 20 or 50 m		

Abbreviation: MR, major road; TSP, total suspended particle; CO, carbon monoxide; NOX, nitrogen oxides; SOX, sulfur oxides; NH3, ammonia; VOC, volatile organic compounds; NDVI, Normalized Difference Vegetation Index; KTDB, Korean Transport Database; KOSIS, Korean Statistical Information Service; SGIS, Statistical Geographic Information Service; EGIS, Environmental Geographical Information Service; ODP, open data portal; SP-IDC, Shipping and Port Integrating Data Center; NSIC, National Spatial Information Clearinghouse; NIER, National Institute of Environmental Research; IIS, Institute of Industrial Science, University of Tokyo; USGS, United States Geological Survey. [a]Different

buffer sizes by category: traffic, 25, 50, 100, 300, 500, and 1000 m; demographic characteristics and land use, 50, 100, 300, 500, 1000, and 5000 m; emissions, 3, 15, and 30 km. [b]Sum of road lengths computed for three methods: single central lines of roads, road lines multiplied by numbers of lanes, and road lines multiplied by numbers of lanes and line widths.

Table S2. Summary statistics of annual average concentrations for $PM_{2.5}$ (μg/m²) in 2010 at 37 regulatory monitoring sites in Seoul, Korea.

	N	Min	Median	Max	Mean	SD
Urban background sites	25	19.8	25.1	27.4	24.9	1.8
Urban roadside sites	12	25.4	30.3	37.0	30.6	3.8
Total	37	19.8	25.8	37.0	26.8	3.7

Table S3. Means and standard deviations of the five selected geographic variables from land use regression of $PM_{2.5}$ annual average concentrations during 2010 across current, subject and candidate locations in Seoul, Korea.

Geographic variables	Current (N = 37) [a]	Subject (31,097) [b]	Candidate (412) [c]
Length of major road * (100m buffer)	112.47 ± 165.67	23.57 ± 71.98	44.92 ± 99.36
Proportion of water surface land use (500m)	0.02 ± 0.06	0.01 ± 0.03	0.01 ± 0.03
Number of construction companies (1000m)	71.15 ± 85.88	48.00 ± 62.64	54.94 ± 68.94
Distance to the nearest bus stop	2.05 ± 0.30	2.11 ± 0.29	1.95 ± 0.35
Number of construction workers (100m)	19.24 ± 31.82	18.83 ± 59.16	32.15 ± 78.99

[a] Current location: regulatory monitoring sites. [b] Subject location: home addresses of the Atopy Free School survey children. [c] Candidate location: community service centers. * Major road defined as all national and metropolitan highways, and local roads with more than six lanes

Table S4. Means and standard deviations of the five selected geographic variables from land use regression of NO_2 annual average concentrations during 2010 across current, subject and candidate locations in Seoul, Korea.

Geographic variables	Current (N = 37) [a]	Subject (31,097) [b]	Candidate (412) [c]
Length of major road multiplied by number of lanes (100m)	765.80 ± 1070.68	137.45 ± 380.64	253.83 ± 512.22
Number of construction (1000m)	3206.12 ± 4176.85	2196.45 ± 2883.40	2445.74 ± 3424.93
Number of buildings constructed before the 1960s (100m)	0.73 ± 1.35	1.06 ± 3.41	1.17 ± 4.29
Proportion of water surface land use (500m)	0.02 ± 0.06	0.01 ± 0.03	0.01 ± 0.03
Proportion of for culture, sports and tourism land use (300m)	0.01 ± 0.03	0.00 ± 0.01	0.00 ± 0.01

[a] Current location: regulatory monitoring sites. [b] Subject location: home addresses of the Atopy Free School survey children. [c] Candidate location: community service centers. *Major road defined as all national and metropolitan highways, and local roads with more than six lanes.

Table S5. Five selected geographic variables and cross-validated R^2s from land use regression of NO_2 annual average concentrations during 2010 in Seoul, Korea.

Variable	β^a	*p* value	CV R^2
Length of major road multiplied by number of lanes (100m)	10.76	<0.001	0.58
Number of employees in construction industries (1000m)	8.08	<0.001	
Number of buildings constructed before the 1960s (100m)	6.85	<0.001	
Proportion of water surface land use (500m)	1.35	<0.001	
Proportion of for culture, sports and tourism land use (300m)	-1.31	0.003	

[a] Estimated regression coefficient multiplied by an increment (90th–10th percentile) of each variable. [b] Major road defined as all national and metropolitan highways, and local roads with more than six lanes.

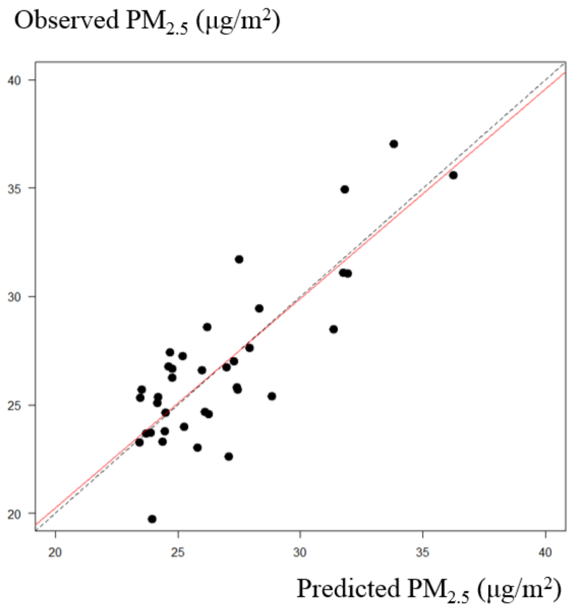

Figure S1. Scatter plot of observed and cross-validation predicted annual average concentrations of $PM_{2.5}$ across 37 regulatory monitoring sites during 2010 in Seoul, Korea (leave-one-out cross-validation R^2 of 0.69).

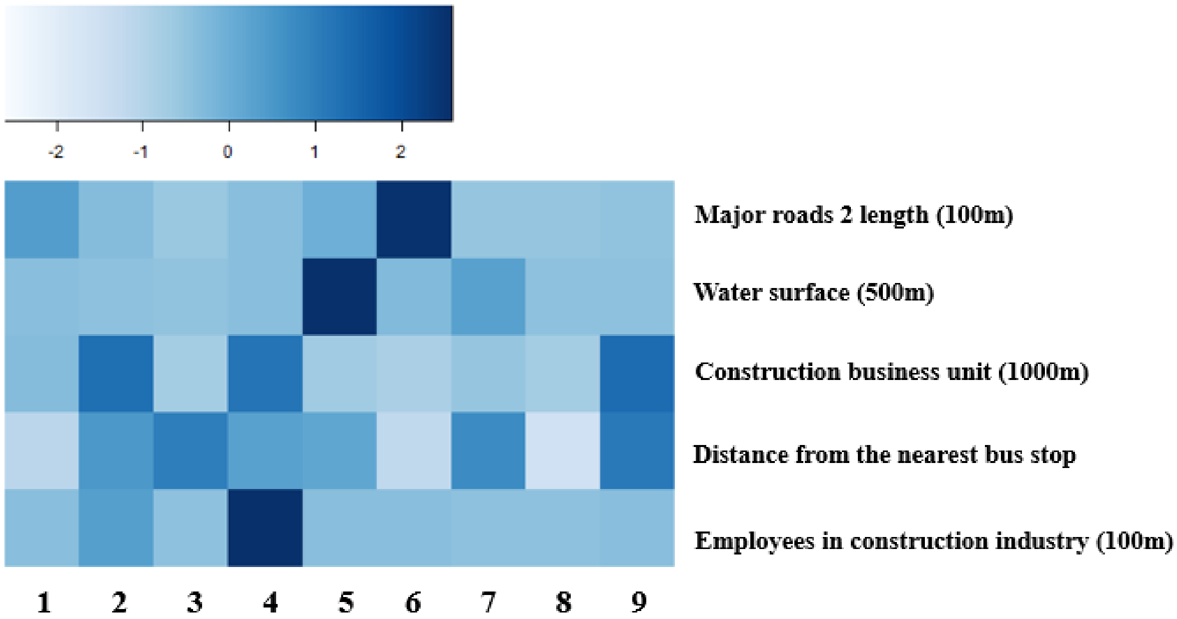

Figure S2. Heatmap of the five geographic variables at current, subject, and candidate locations across nine clusters for $PM_{2.5}$.

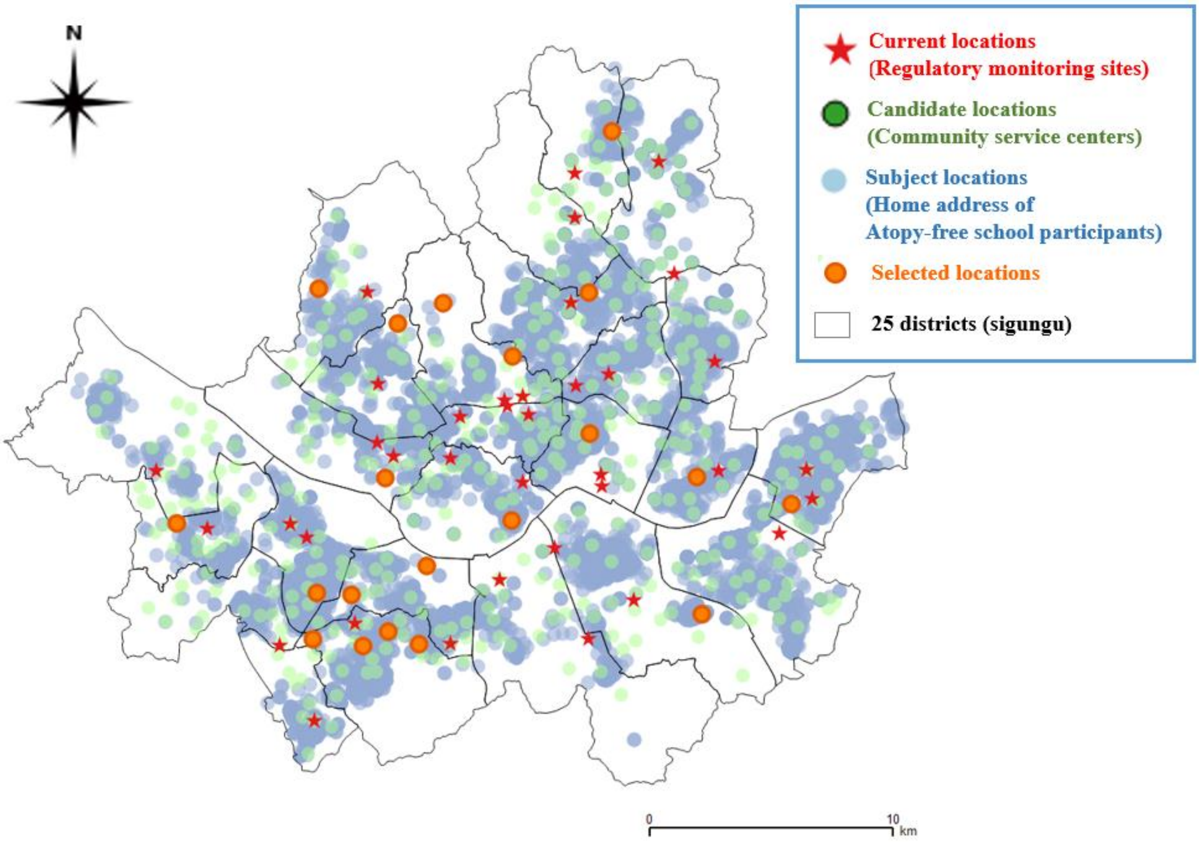

Figure S3. Map of 20 selected new monitoring sites for $PM_{2.5}$ along with current, candidate, and subject locations in Seoul, Korea.

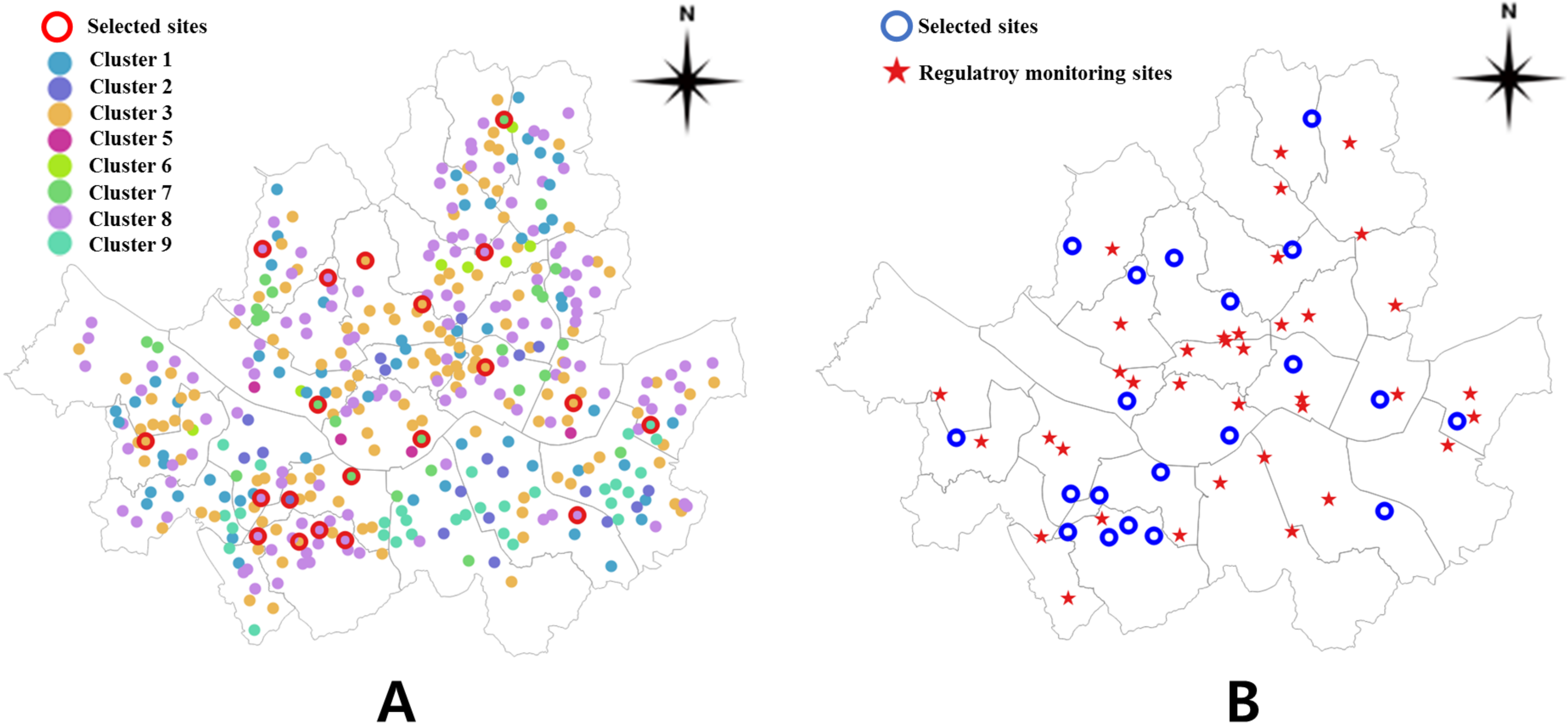

Figure S4. Maps of candidate sites in each of the nine clusters determined from cluster analysis for $PM_{2.5}$ (**A**) and new selected sites with regulatory monitoring sites (**B**).

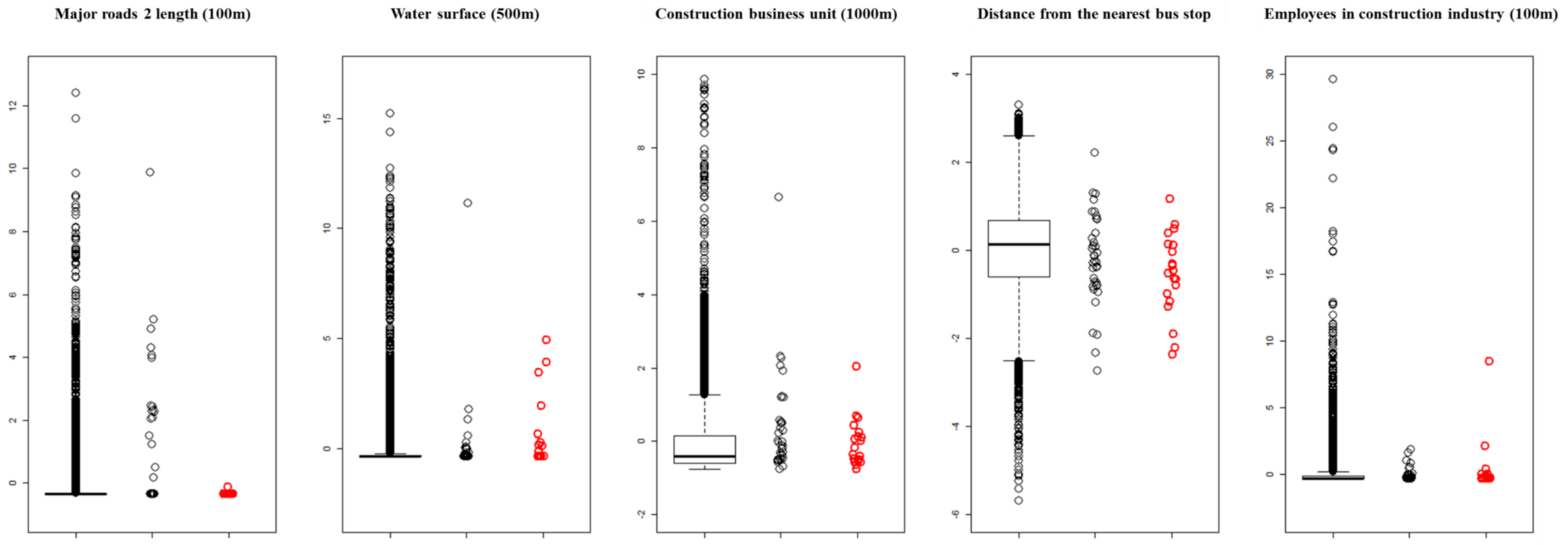

Figure S5. Distributions of five scaled geographic variables across subject locations (left), current locations (middle), and new sites (right) selected from candidate locations by using the monitoring design for $PM_{2.5}$.

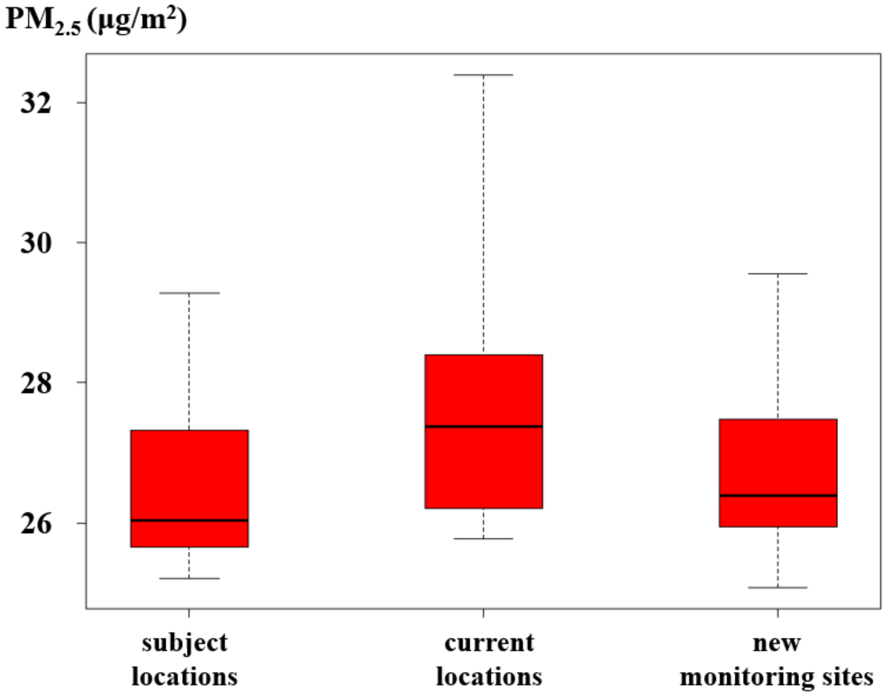

Figure S6. Variability of predicted $PM_{2.5}$ across subject locations (left), current locations (middle), and new sites (right).

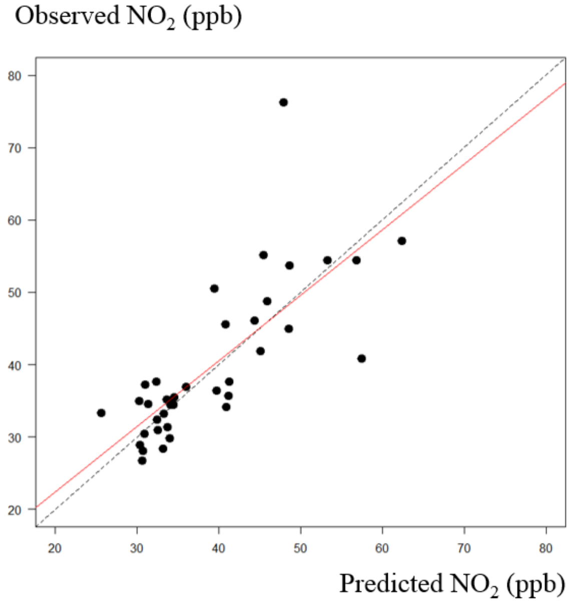

Figure S7. Scatter plot of observed and cross-validation predicted annual average concentrations of NO_2 across 37 regulatory monitoring sites during 2010 in Seoul, Korea (leave-one-out cross-validation R^2 0.58).

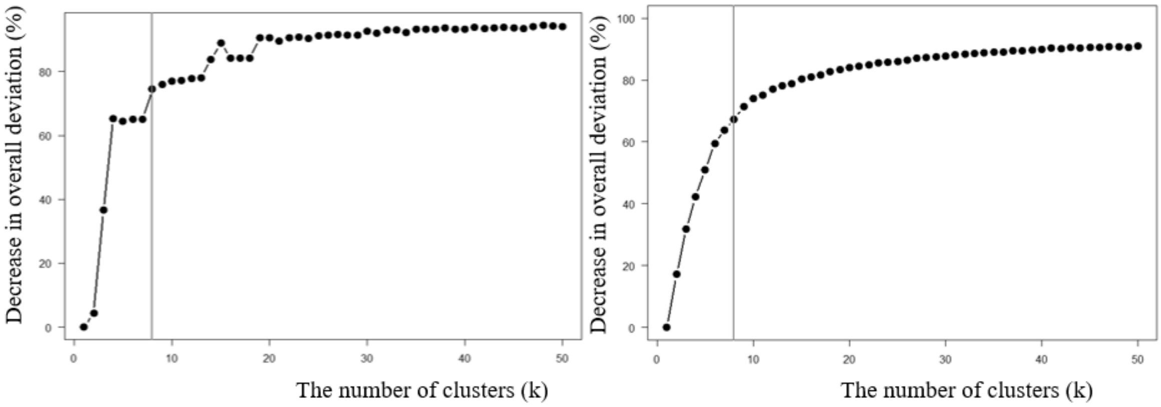

Figure S8. Decrease in overall deviation (DiD) based on predicted NO_2 concentration (left) and five geographic variables (right) against the numbers of clusters (k) (vertical lines indicating nine clusters).

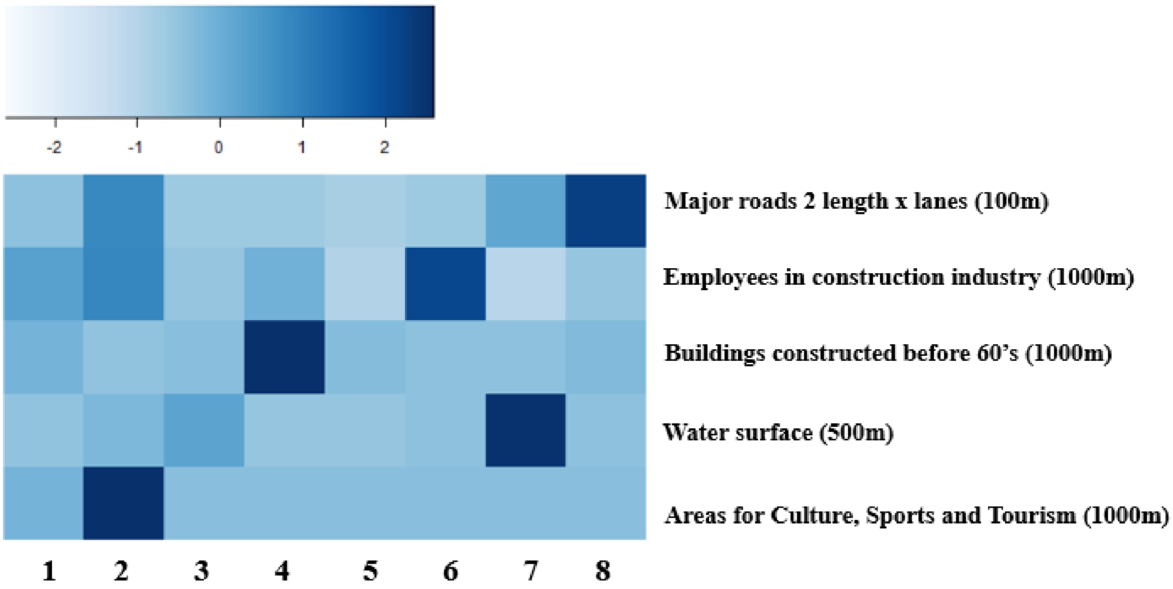

Figure S9. Heatmap of the five geographic variables at current, subject, and candidate locations across eight clusters for NO_2.

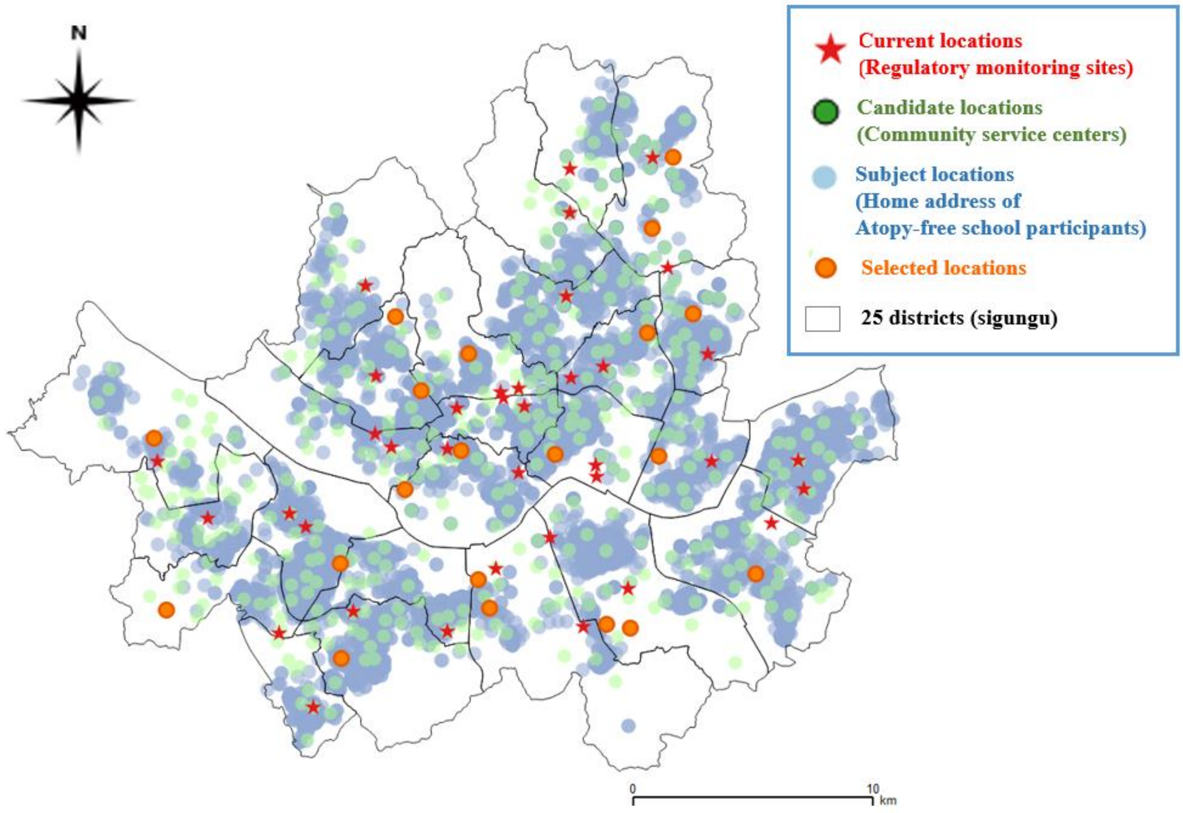

Figure S10. Map of 20 selected new monitoring sites for NO_2 along with current, candidate, and subject locations in Seoul, Korea.

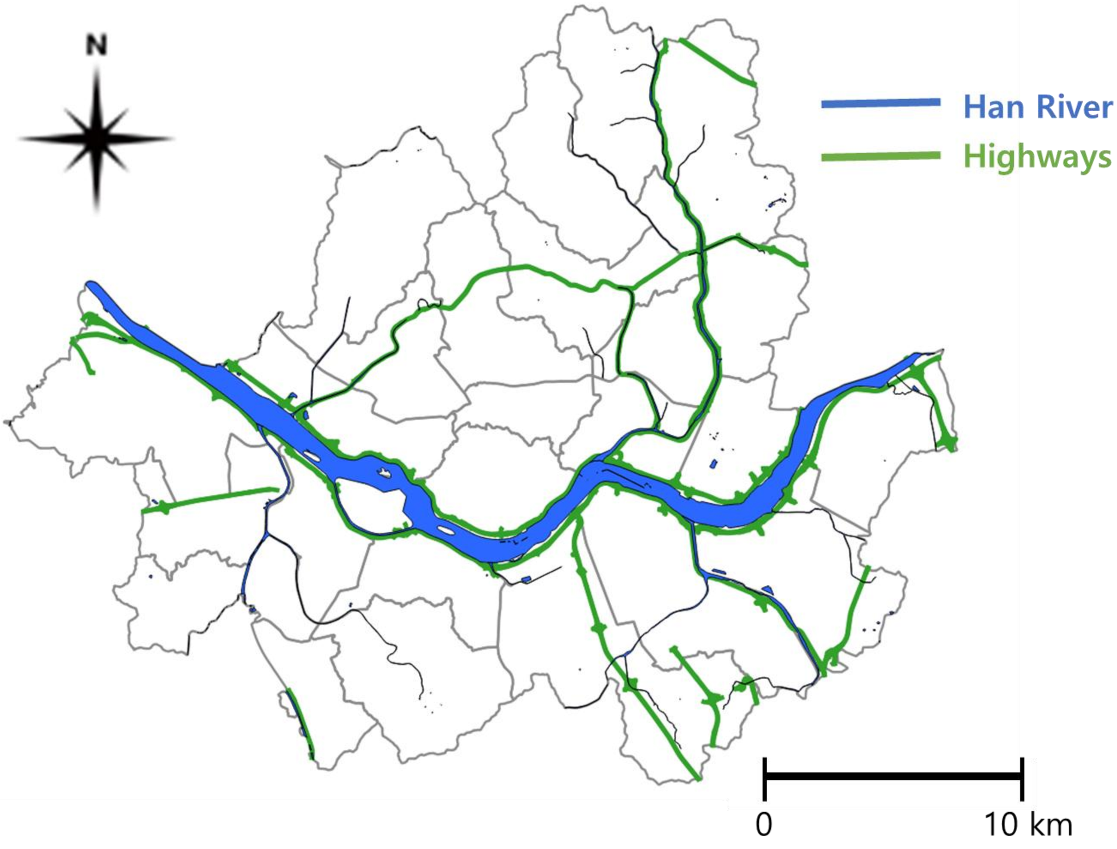

Figure S11. Map of Han River and metropolitan and national highways in Seoul.

© 2017 by the authors; licensee MDPI, Basel, Switzerland. This article is an open access article distributed under the terms and conditions of the Creative Commons by Attribution (CC-BY) license (http://creativecommons.org/licenses/by/4.0/).
